# Peripheral Injection of hUC-MSCs in the Treatment of Acute Liver Failure: A Pre-Clinical Cohort Study in Rhesus Monkeys

**DOI:** 10.1155/2024/4654912

**Published:** 2024-07-16

**Authors:** Yuting Zeng, Zhenru Wu, Gen Chen, Guoqiang Liu, Bo Zhang, Yongjie Zhou, Menglin Chen, Rong Yao, Yujun Shi

**Affiliations:** ^1^ Liver Transplant Center Transplant Center and Key Laboratory of Transplant Engineering and Immunology NHC West China Hospital Sichuan University, Chengdu, China; ^2^ Institute of clinical Pathology West China Hospital Sichuan University, Chengdu, China; ^3^ Development and Application of Human Major Disease Monkey Model Key Laboratory of Sichuan Sichuan Yibin Horizontal and Vertical Biotechnology Co., Ltd., Yibin 644601, China; ^4^ Sichuan Stem Cell Bank and Sichuan Neo-Life Stem Cell Biotech Inc., Chengdu 610037, China; ^5^ Laboratory of Liver Transplantation West China Hospital Sichuan University, Chengdu 610041, China; ^6^ Department of Emergency Medicine Emergency Medical Laboratory West China Hospital Sichuan University, Chengdu, Sichuan, China

## Abstract

**Background:**

Using a toxin-induced lethal acute liver failure (ALF) monkey model, we have recently shown that early peripheral infusion of human umbilical cord mesenchymal stem cells (hUC-MSCs) can alleviate liver damage and improve animal survival by suppressing the activation of circulating monocytes and the subsequent cytokine storm. Here, we explored whether the administration of hUC-MSCs could still improve ALF when the cytokine storm is fully developed.

**Method:**

We treated ALF monkeys with peripheral delivery of hUC-MSCs at 48 hr after toxin challenge. Liver indices, histology, imaging, and animal survival were recorded and analyzed.

**Results:**

In our cohort study, we conducted and demonstrated that the infusion of hUC-MSCs significantly improved liver histology, effectively controlled inflammatory cytokine storms, and increased survival rates. Additionally, the administration of a higher dose of hUC-MSCs (2 × 10^7^/monkey) yielded superior outcomes compared to a lower dose (1 × 10^7^/monkey).

**Conclusion:**

Treatment of hUC-MSCs can significantly improve the pathological and survival outcomes of ALF even when the cytokine storm has been fully developed, indicating a promising clinical solution for ALF.

## 1. Introduction

Acute liver failure (ALF) [1] is characterized by rapid deterioration of hepatic function resulting from hepatocyte damage caused by various etiological factors, such as infection with hepatitis virus, major hepatectomy, toxic drugs, and nonpharmacological intoxication (e.g., mushroom poisoning). Severe liver injury-induced coagulopathy and altered mention, namely, hepatic encephalopathy, are required for the diagnosis of this rare and life-threatening clinical syndrome, according to the European Association for the Study of the Liver (EASL) [2]. Hepatocyte transplantation (HCT) and liver transplantation (LT) are regarded as effective therapies for ALF. However, HCT has limitations, such as an unknown source of hepatocytes, a high demand for cells, and the inability to proliferate in vitro. Meanwhile, LT is unavailable for most patients due to the shortage of donor livers and the limited therapy window. A reported 71%–85% of 1-year survival rate was observed in patients who underwent emergency LT [3, 4], while 82% of ALF patients died within a week of diagnosis while awaiting an organ [3]. Thus, new feasible and reliable alternative treatments are greatly needed to improve the outcomes of ALF patients.

Amanitin-containing species are responsible for over 90% of fatalities caused by mushroom intoxications worldwide [5], and ALF is the featured clinical syndrome in amanitin-poisoned patients. In particular, *α*-amanitin is believed to be the main cause of ALF by leading to hepatocyte dysfunction and, ultimately, cell death through inhibition of polymerase II and protein synthesis [6]. In addition, the reabsorption of *α*-amanitin via the enterohepatic circulation further prolongs and exacerbates the hepatotoxic effect [7].

In our previous work, low-dose *α*-amanitin extracted from *Amanita phalloides* (AP; “death cap”) and lipopolysaccharide (LPS) were used to establish rhesus monkey models of ALF [8]. Although a mouse model of ALF has been used to study the related mechanism and potential medical therapy [9], our large primate model more effectively simulated human cases with the similar metabolic and physiopathological process. The monkeys exhibited typical symptoms and physiopathological changes observed in ALF cases, including dynamic changes in serology, histology, imaging, and clinical behaviors. Based on monkey models, we uncovered the essential role of activated circulating monocytes (c-Mos) in initiating ALF rather than Kupffer cells that are specific to the liver [10, 11]. A broad screening of serology and cytokines indicated that activated monocytes were critical in the development and exacerbation of hepatocyte injuries through massive secretion of interleukin 6 (IL-6). The subsequent excessive systemic inflammation triggered by IL-6 was the main factor promoting hepatocyte necrosis, loss of systemic homeostasis, and eventual fatality. Our findings revealed that c-Mos were a promising therapeutic target, and its activation should be inhibited prior to uncontrolled systemic inflammation.

Mesenchymal stem cells (MSCs), a group of cells with the ability to self-renew and differentiate, garnered significant interest over the past decade due to their therapeutic potential in various diseases [12, 13, 14]. In particular, human umbilical cord mesenchymal stem cells (hUC-MSC) [15, 16, 17] and hUC-MSC-derived exosomes [18] have shown protective effects against acute hepatic injury, fibrosis, and other disorders. We recently demonstrated that peripheral delivery of hUC-MSCs significantly improved the survival of monkeys with ALF [19]. Our previous report [10] identified that IL-6 secreted by c-Mos was the key initiator of the inflammatory cascade, and hUC-MSCs simultaneously suppressed the activation of c-Mos and its aggregation in the liver [19]. The monkeys treated with hUC-MSCs had profoundly ameliorated clinical manifestations, physiopathology, and serology. The suppression of c-Mos possibly resulted from the inhibitory miRNA in the exosome released by hUC-MSCs after toxin stimulation [18], which involved factors that inhibited the inflammatory reaction and immune response. This finding suggests that hUC-MSCs and their derivatives may be potential therapeutic options for ALF and other liver disorders.

Compared to many other tissues and organs, the human umbilical cord is a rich source of MSCs without risk to donors, and hUC-MSCs possess a great capacity for proliferation and differentiation into hepatocyte-like cells [20]. The low immunogenicity of hUC-MSCs allows for allogeneic transplantation and even xenogeneic transplantation, both of which have been demonstrated to be feasible in monkeys [19, 21]. Moreover, we did not detect carcinogenesis in the recipient monkeys during follow-ups of over 5 years. Therefore, hUC-MSC-based treatment is anticipated to be a safe, feasible, and effective solution for ALF that meets the prerequisites for translation to clinical application. In this study, we conducted a cohort study to further validate the efficacy and safety of hUC-MSCs in the rhesus monkey model of toxin-induced ALF.

## 2. Results

### 2.1. Peripheral Infusion of hUC-MSCs Significantly Improves Survival in Monkeys with ALF

**Figure 1:**
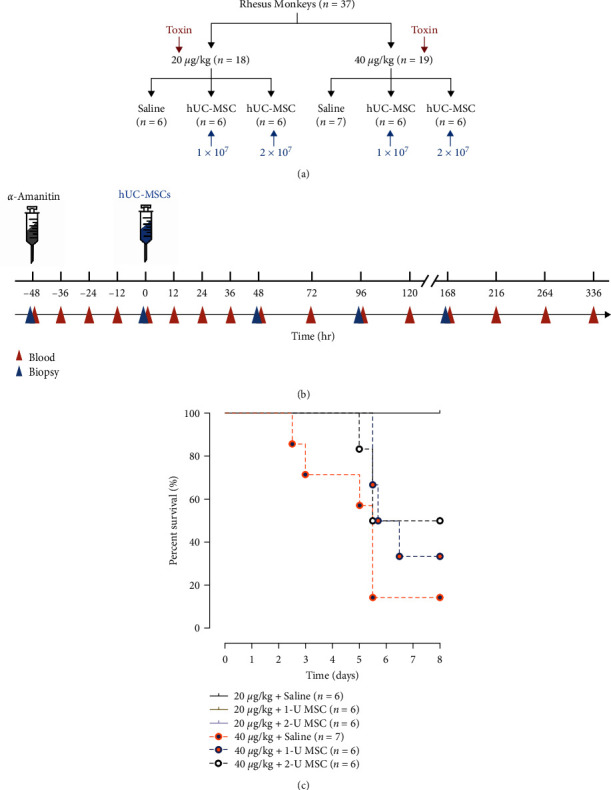
hUC-MSCs improve survival in monkeys with ALF. (a) Schematic representation of the experimental grouping. (b) Schematic representation of the experimental design. (c) Survival curves for the monkeys submitted to different treatments (Kaplan–Meier method with log-rank test) *P* < 0.001.

For the establishment of the toxin-induced ALF model, a total of 37 monkeys were recruited and randomly divided into two dose groups: low dose (*α*-amanitin at 20 *μ*g/kg, *n* = 18) and high dose (*α*-amanitin at 40 *μ*g/kg, *n* = 19). After the toxin infusion, the monkeys in each dose group were further randomly assigned to three subgroups, receiving either 1 U or 2 U of hUC-MSCs or an identical volume of saline at 48 hr post *α*-amanitin infusion (Figure 1(a)). Liver biopsies and blood collection were conducted at indicated time points (Figure 1(b)).

Nearly all monkeys demonstrated progressive poor appetite, mental indifference, and fatigue 24–48 hr after toxin injection. The clinical symptoms and signs were more common and severe in animals injected with a high dose of *α*-amanitin. The monkeys were euthanized when an irreversible hepatic coma developed. All 18 monkeys who received a low dose of *α*-amanitin survived, regardless of treatment. While no monkey that received a high dose of *α*-amanitin died before 48 hr following toxin exposure, six (85.7%), four (66.7%), and three (50.0%) monkeys died within the first week, with median survival times of 68, 86, and 108 hr in the saline, 1-U and 2-U hUC-MSC-treated group, respectively. The hUC-MSC therapy obviously improved the overall survival of animals with severe ALF (*P*=0.036; Figure 1(c) and Additional file 1: Table S1). All the monkeys that survived for the first week have achieved long-term survival and have been followed up for 5 years so far.

### 2.2. hUC-MSCs Improve Liver Histology and Hepatic Indices

**Figure 2:**
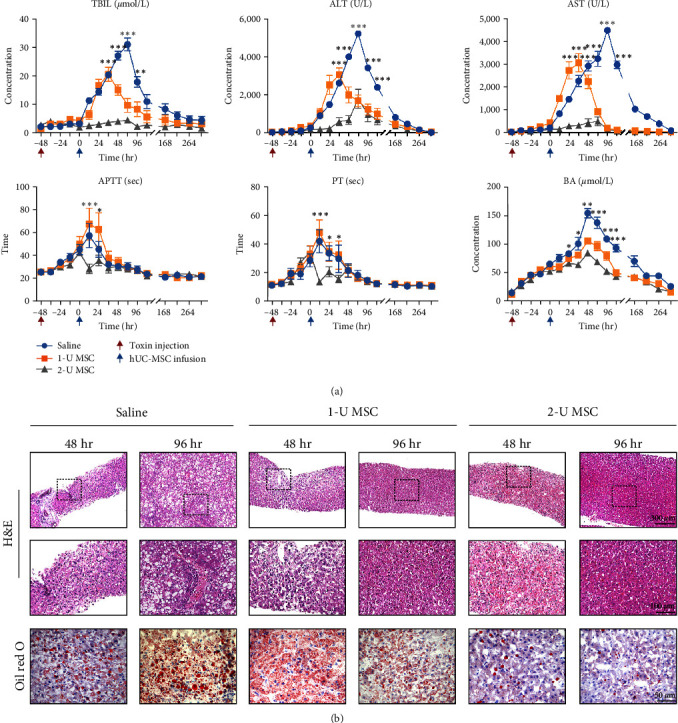
Peripheral delivery of hUC-MSCs ameliorates liver histology and hepatic indices in 20 *μ*g/kg of *α*-amanitin group. (a) Biochemical assay of hepatic indices: alanine aminotransferase (ALT), glutamic-oxaloacetic transaminase (AST), total bilirubin (TBIL), prothrombin time (PT), activated partial thromboplastin time (APTT), and blood ammonia (BA). Error bars, SEM.  ^*∗*^*P* < 0.05,  ^*∗∗*^*P* < 0.01, and  ^*∗∗∗*^*P* < 0.001, compared with the data at 48 hr. (b) HE staining and Oil red O staining of liver biopsy at indicated times.

In spite of the fact that all the monkeys survived when they received a low dose of amanitin injection, a significant increase in most hepatic indices, including bilirubin and liver enzymes, was observed 48 hr after toxin exposure. In saline-treated monkeys, these indices began to soar and peaked at 72 or 96 hr (Figure 2(a)). A similar variation was observed in 1-U hUC-MSC-treated monkeys, but all their parameters showed lower peak values and began to decrease earlier than in the saline group. In addition, monkeys treated with 2 U of hUC-MSCs displayed much lower peak values even than those in 1-U hUC-MSC-treated monkeys. We observed a 14-fold increase in the peak value of total bilirubin (TBIL) to 31 *μ*mol/L in the saline-treated group, which decreased to 20 and 4.5 *μ*mol/L after different doses of hUC-MSC intervention. More incredible fold changes were also observed in alanine aminotransferase (ALT) and aspartate aminotransferase (AST), a 186-fold increase in ALT to 5,211 U/L and a 119-fold increase in AST to 4,460 U/L. Then ALT decreased to 3,058 U/L in the 1-U hUC-MSC group and 1,807 U/L in the 2-U hUC-MSC group, while AST decreased to 3,053 U/L and 508 U/L in the two groups, respectively. Likewise, saline-treated monkeys showed the worst coagulative function evidenced by prothrombin time (PT) and activated partial thromboplastin time (APTT), which reached 57 s and 42 s, respectively. Blood ammonia (BA) kept growing after toxin infusion in all 18 monkeys, peaking at 154 *μ*mol/L 48 hr after hUC-MSC injection and then gradually declining to normal level (Figure 2(a)). Monkeys who received 1-U hUC-MSCs had higher BA than those who had 2-U hUC-MSCs but lower than those without access to stem cell therapy throughout the first 2 weeks. The five indices of direct bilirubin (DBIL), indirect bilirubin (IBIL), alkaline phosphatase (ALP), glutamyl transpeptidase (GGT), and globulin (GLO) were significantly lower in the hUC-MSC-treated groups (1 U and 2 U) compared to the saline-treated group (Figure S1).

The biopsies at 48 hr after toxin exposure showed mild to severe steatosis, focal necrosis, and infiltration of inflammatory cells in the liver. Then another 48 hr later, hUC-MSC therapy seemed to more noticeably improve the histology compared to saline-treated ones, although the histology of each group recovered to some extent (Figure 2(b)).

**Figure 3:**
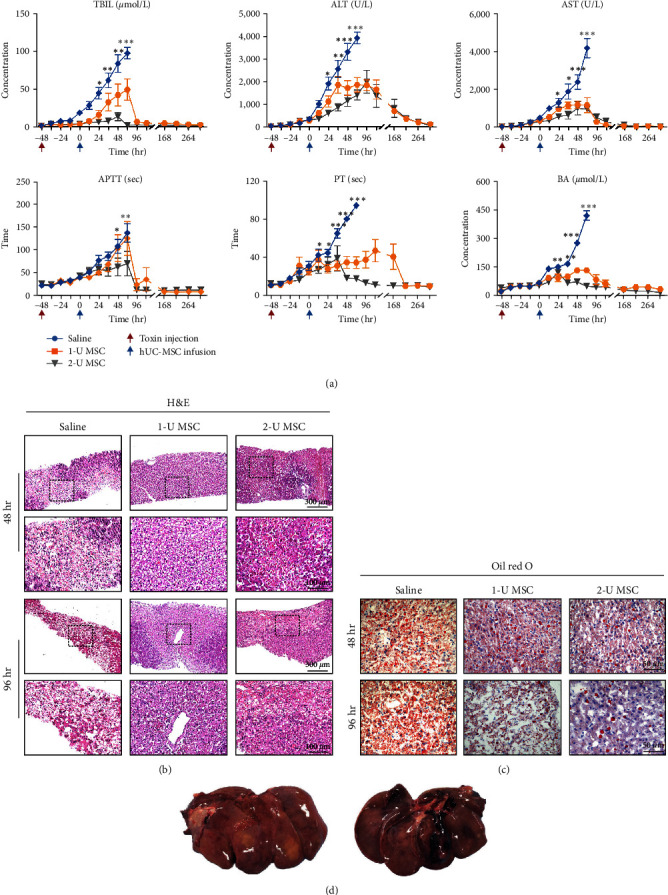
Peripheral delivery of hUC-MSCs ameliorates liver histology and hepatic indices in 40 *μ*g/kg of *α*-amanitin group. (a) Biochemical assay of hepatic indices: alanine aminotransferase (ALT), glutamic-oxaloacetic transaminase (AST), total bilirubin (TBIL), prothrombin time (PT), activated partial thromboplastin time (APTT), and blood ammonia (BA). Error bars, SEM.  ^*∗*^*P* < 0.05,  ^*∗∗*^*P* < 0.01, and  ^*∗∗∗*^*P* < 0.001, compared with the data at 48 hr. (b) Gross and histopathological changes in a severe ALF liver. (c) Oil red O staining of liver biopsy at indicated times. (d) HE staining of liver biopsy at indicated times.

After a high dose of amanitin challenge, the monkeys without hUC-MSC infusion exhibited a sharp and dramatic increase in hepatic indices. All the indices peaked at around 120 hr, reaching hundreds of times the normal upper limit (Figure 3(a)). TBIL increased 42-fold to 97 *μ*mol/L, ALT increased 72-fold to 3,917 U/L, and AST increased 110-fold to 4,190 U/L. Because only one monkey survived, we did not analyze its parameters further. Infusion of hUC-MSCs profoundly ameliorated the hepatic indices; all the parameters showed much lower peaks at a similar time point to saline-treated monkeys and then gradually returned to normal levels with 1–2 weeks (Figure 3(a)). TBIL decreased to 49.5 *μ*mol/L in 1-U hUC-MSC-treated monkeys and 14.12 *μ*mol/L in 2-U hUC-MSC-treated monkeys; ALT decreased to 1,860 U/L and 1,994 U/L in monkeys treated with two different doses of hUC-MSCs, respectively; and AST decreased to 1,202 and 1,015 U/L. The five indices of DBIL, IBIL, ALP, GGT, and total bile acid (TBA) were significantly lower in the hUC-MSC-treated groups (1 U and 2 U) compared to the saline-treated group (Figure S2).

The biopsy showed that 48 hr after the high dose of amanitin challenge, the livers developed mild-to-moderate steatosis and focal necrosis. Then, lethal patchy necrosis and sinusoid hyperemia appeared at later times if hUC-MSC infusion was unavailable. For monkeys that received hUC-MSC therapy, hepatic histology also showed apparent steatosis, piecemeal necrosis, and infiltration of inflammatory cells (Figures 3(b) and 3(c)). The autopsy of the deceased monkeys showed a shrunken appearance and visible ecchymosis on the surface of the failed liver (Figure 3(d)). However, the treatment succeeded in remarkably alleviating hepatic necrosis and bleeding and inhibiting the disintegration of hepatic lobules.

### 2.3. hUC-MSCs Inhibit the Activation of Circulating Monocytes and Suppress Systemic Inflammation

**Figure 4:**
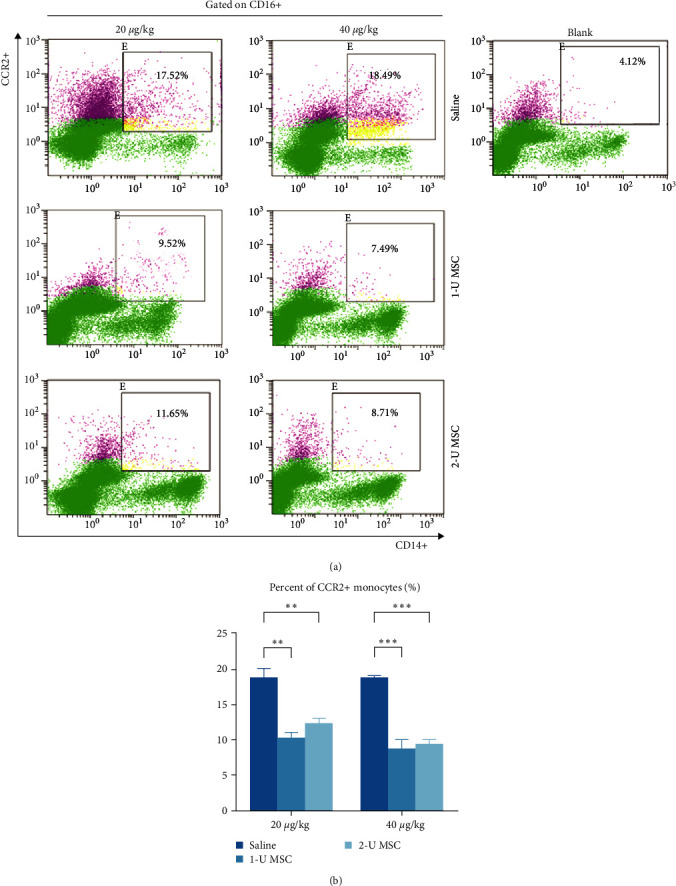
hUC-MSCs inhibit the activation of circulating monocytes. (a and b) Flow cytometry analysis of activated monocytes (CD14+ CD16+ CCR2+) in subsets of peripheral blood monocytes. Error bars, SEM, Student's *t*-test,  ^*∗∗*^*P* < 0.01, and  ^*∗∗∗*^*P* < 0.001.

Our previous study showed that c-Mos are critical in accelerating ALF by producing IL-6. Early infusion of hUC-MSCs could inhibit the activation of c-Mos and thereby prevent the liver from excessive inflammatory injury [19]. We explored whether the infusion of hUC-MSCs has a role in regulating c-Mos activation and systemic inflammation when c-Mos and a cytokine storm were robustly activated. Flow cytometry assay revealed that the proportion of CD14+ CD16+ CCR2+ monocytes in peripheral blood was approximately halved at 24 hr after hUC-MSC intervention (Figures 4(a) and 4(b)).

**Figure 5:**
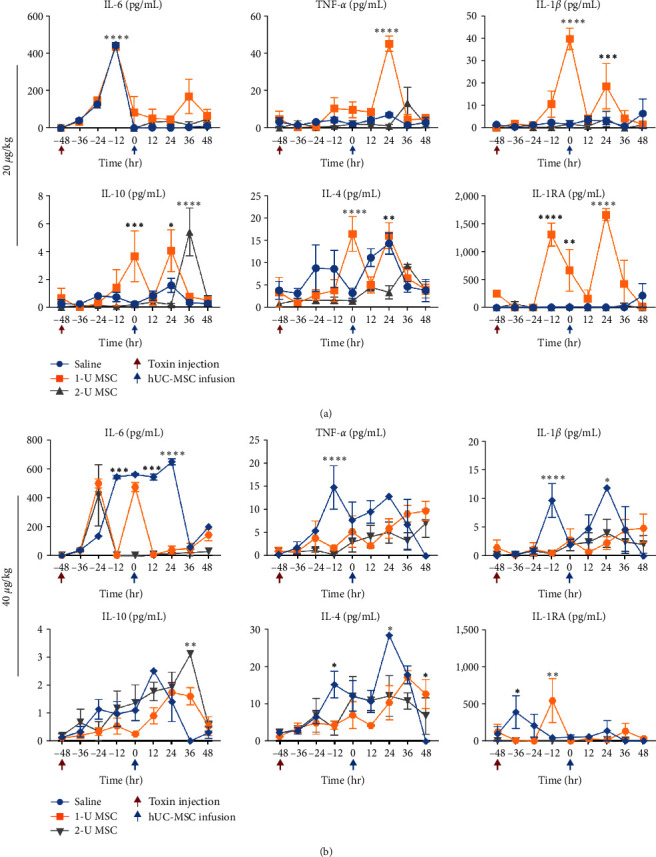
hUC-MSCs suppress systemic inflammation. (a and b) Show the serum levels of cytokines including TNF-*α* (tumor necrosis factor), IL-6, IL-1*β*, IL-10, IL-4, and IL-1RA (IL-1 receptor antagonist) in response to 20 *μ*g/kg and 40 *μ*g/kg of *α*-amanitin challenge, respectively. Error bars, SEM.  ^*∗*^*P* < 0.05,  ^*∗∗*^*P* < 0.01,  ^*∗∗∗*^*P* < 0.001, and  ^*∗∗∗∗*^*P* < 0.0001, compared with the data at −48 hr.

IL-6 is considered both an inflammatory cytokine and a regenerative mediator in liver conditions. The secretion of IL-6 in ALF affects the complete recovery and reconstitution of liver parenchyma and liver function. In animals injected with either dose of toxin, IL-6 began to increase at 12–24 hr. Additionally, other circulating inflammatory factors, such as TNF-*α* and IL-1*β*, showed similar trends. They peaked between 24 and 48 hr after toxin induction. Although the fluctuation of data might have impaired the statistical difference, we could see that hUC-MSC infusion played a role in suppressing the serum levels of inflammatory cytokines but increasing some anti-inflammatory and protective factors, such as IL-10, IL-4, and IL-1RA. This effect was more obviously observed in monkeys that received a high dose of toxin. Six out of seven saline-treated monkeys were sacrificed without a noticeable decline in these cytokines, while it took the only surviving monkey another week to control systemic inflammation. hUC-MSCs, particularly in a higher concentration, remarkably ameliorated systemic disarrangement around 48 hr after infusion (Figure 5).

### 2.4. hUC-MSC Infusion Facilitates Liver Regeneration

**Figure 6:**
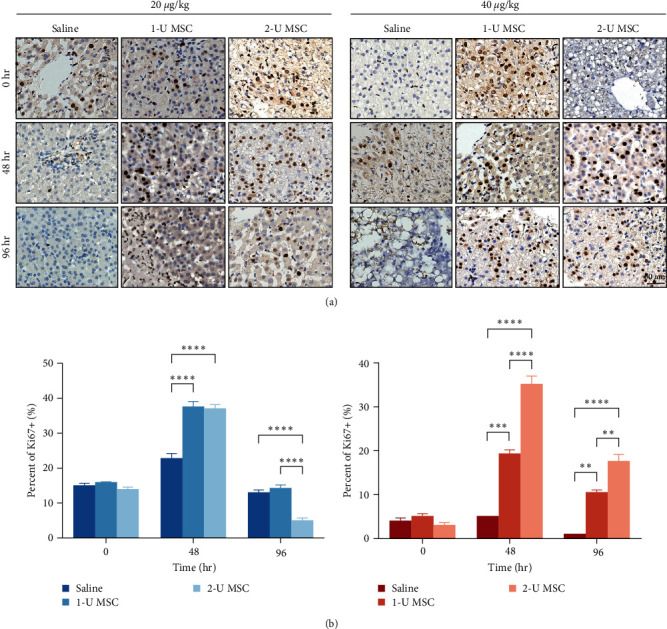
hUC-MSC infusion promotes liver regeneration. (a) Ki67 immunohistochemical staining of liver specimens at indicated times after infusion of hUC-MSC. (b) Quantitation of Ki67+ positive hepatocytes at indicated times following different treatments. The numbers of positive cells in 10 consecutive high-power fields were counted. Error bars, SEM, Student's *t*-test,  ^*∗∗*^*P* < 0.01,  ^*∗∗∗*^*P* < 0.001, and  ^*∗∗∗∗*^*P* < 0.0001.

Active liver regeneration, indexed by Ki67 immunohistochemistry staining, was observed in all monkeys during the first 4 days after toxin injection, except that very rare proliferating hepatocytes were detected in the autopsied livers. Notably, infusion of hUC-MSCs enhanced the Ki67 indexes two- to threefold at each indicated time point (Figures 6(a) and 6(b)). Proliferation was detectable up to 4 days after toxin injection in all survived monkeys. Interestingly, the only surviving monkey treated with saline experienced a delayed but significantly more prolonged regeneration period than those treated with hUC-MSCs.

### 2.5. Monkeys That Survived ALF Remain Healthy in the Long-Term Follow-Ups

To further confirm the safety of hUC-MSC transplantation, clinicopathologic and imaging evaluations were applied in the six surviving monkeys every year for 5 years. Most serum parameters were kept at normal levels, including blood counts, hepatic indices, renal function, and clotting times. No tumoral lesion, fibrosis, or thrombosis was detected in ultrasonography, MRI, or liver biopsies. In addition, no immune disorders, including those related to immune cells, antibodies, and complements, were detected. Our results demonstrated that hUC-MSC treatment was not associated with carcinogenesis or other secondary complications.

## 3. Discussion

There is still no robust nonsurgical strategy for ALF, partially due to its unclear pathophysiological mechanism. In the current study, we established primate animal models of ALF using amanitin alone for the first time to further evaluate the therapeutic effect of hUC-MSCs. Compared to our previous preliminary study, the following modifications were made for certain reasons: (1) Only *α*-amanitin was used to establish the ALF model, rather than a combination of *α*-amanitin and LPS [8, 10]. All saline-treated animals died after infusion of *α*-amanitin and LPS in previous studies [19, 22], where the mortality was significantly higher than in a clinical setting [7]. Besides, the intraperitoneal injection of LPS was supposed to activate peritoneal macrophages before being absorbed by the peritoneum and entering circulation [23], making it extremely difficult to determine the role of the c-Mos in ALF pathogenesis. The addition of LPS might result in a misleading conclusion. (2) Early hUC-MSC therapy is critical, as c-Mos-derived IL-6 increases dramatically within 24 hr after the toxin challenge [10]. We previously treated monkeys with hUC-MSCs only 2 hr after toxin injection. However, human cases caused by a variety of etiological factors, not limited to poisonous mushroom intake, are often admitted with apparent manifestation after the full development of symptoms and signs. Here, we set out to explore whether hepatic indices and prognosis could be improved by hUC-MSCs at 48 hr after toxin injection when the cytokine storm is fully developed. (3) Preliminary experiments suggested that an increased number of hUC-MSCs was more effective in improving liver histology and hepatic function. In the current study, an infusion of 1 × 10^7^ (1 U) or 2 × 10^7^ (2 U) stem cells per monkey was applied to reveal the dose effect of hUC-MSCs, which was ambiguous before. (4) Last but not least, we explored the potential role of hUC-MSCs in ameliorating cytokine storm when the c-Mos are fully activated. We found that hUC-MSC-treatment significantly improved toxin-induced hepatic indices, liver histology, systemic inflammation, and survival.

In our previous work, all monkeys who received a lethal dose of toxins were rescued by immediate peripheral infusion of hUC-MSCs, which prevented the activation of c-Mos and IL-6 secretion and the secondary cytokine storms [19]. However, in routine clinical settings, symptoms of ALF from various causes, such as initial jaundice, lethargy, and mental alterations, are hardly noted, and the best and most effective time for treatment is always missed [2]. It is, therefore, worth exploring whether there is still a role for hUC-MSC therapy when c-Mos have been already fully activated [2]. Herein, our study suggested that delayed hUC-MSC treatment could also significantly improve the liver indices and histology in nonfatal ALF and survival outcomes of monkeys with fatal ALF. We noted that a doubled dose of hUC-MSC therapy was related to an earlier recovery from ALF in surviving monkeys according to earlier ameliorated liver function, serum BA, and clinical manifestations. Although neither the survival rate (50.0% vs. 33.3%) nor the median survival time (108 hr vs. 86 hr) was significantly improved in animals with severe ALF treated with 2-U hUC-MSCs compared with those that received 1-U cell therapies, though tendencies were observed. It is undoubtedly that the efficiency of MSC therapy is determined by their potential and infused cell number. Our data recommend a high dose of cell infusion if available.

The survival benefit from hUC-MSCs in ALF models was exerted mainly through an inhibitory effect on the activation and proliferation of immune cells and severe acute inflammation [16, 19]. Though hepatocyte injury is the initial event, it is widely believed that uncontrolled immune-driven inflammation in response to liver damage is central to the pathogenesis of liver and extrahepatic organ failures [10, 24, 25]. In ALF, systemic inflammation results in hepatic dysfunction, thereby leading to diminished ammonia detoxification and hyperammonemia, which is directly associated with hepatic encephalopathy and ALF-related mortality [26]. As for the therapeutic effect of hUC-MSCs, their multiple immunomodulatory properties are involved: (1) suppressing proliferation or activation of immune cells, such as peripheral blood mononuclear cells (PBMCs) [27] and monocytes [19]; (2) inhibiting expression of pro-inflammatory factors, such as TNF-*α*, IL-1*β*, IL-6, and IL-8, thereby controlling inflammation and hepatocyte apoptosis [19, 28]; and (3) promoting secretion of anti-inflammatory cytokines [15, 19], such as IL-4 and IL-10, which prevent excessive inflammation. Although we infused hUC-MSCs when the c-Mos were activated and the cytokine storm was inflamed, the number of activated c-Mos and the serum levels of proinflammatory factors could be decreased. Our findings further support the immunoregulatory role of hUC-MSCs played in ALF, and delayed cell infusion is also recommended to ameliorate sustained overactivation of inflammatory cells or promote the secretion of inhibitory factors.

Apart from the protective effect on liver function and hepatocyte necrosis, we suspect that hUC-MSCs were associated with more activated hepatocyte proliferation and shortened time required for the completion of liver repair in surviving monkeys. After a steady rise in the first 2 or 3 days, proliferation in the saline-treated animals showed a sharp decline along with a phenomenal increase in inflammatory cytokines. In contrast, reparative ability continued to increase after hUC-MSC administration before a gradual decrease in the survived monkeys. Immune cells exert dual roles at different phases of ALF, from promoting to inhibiting hepatocyte proliferation [29]. TNF-*α*, IL-1, and IL-6 initially generated by liver-specific macrophages (KCs) were found to induce liver proliferation in the early stage in response to liver injury [30]. Furthermore, circulating macrophages also contributed to hepatocyte regeneration and vascularization [31, 32]. However, the subsequent systemic inflammation due to the massive activation of macrophages disrupted hepatic homeostasis and parenchymal repair [22], which was ameliorated by hUC-MSC therapy in our study.

As xenogeneic cells in preclinical settings, hUC-MSCs were well-tolerated in our primate models [19]. hUC-MSC activation was not supposed to result in allogeneic immune rejection according to their minimal expression of human leukocyte antigen DR (HLA-DR) after activation [27]. hUC-MSCs have been increasingly used to treat a variety of human diseases, for example, systemic lupus erythematosus (SLE) [33], arthritis [34], spinal cord injury (SCI) [35], and severe COVID-19 [35]. All these instances demonstrated the long-term safety and efficacy of hUC-MSC therapy. Another safety concern of translating hUC-MSC therapy to clinical practice is the potential for tumorigenicity. Previous studies reported no tumors either in vitro or in mice [36, 37]. Likewise, none of our monkeys developed tumor with follow-up of more than 5 years. Therefore, based on current evidence, hUC-MSC transplantation is expected to be safe.

## 4. Materials and Methods

### 4.1. Experimental Animals

All experimental protocols were approved by the Animal Care and Use Committee of West China Hospital of Sichuan University (Approval No. 2018110A) and met institutional and national guidelines. Feeding and management were carried out in accordance with the regulations of Medical Laboratory Animals of Sichuan Province and Sichuan University. Male adult experimental rhesus monkeys aged 4–6 years were purchased from Green-house Biotech Co., Ltd. (license no.: SCXK (CHUAN) 2014-013, Chengdu, China). Details of the monkeys and their management are available in the supplemental information.

### 4.2. Animal Anesthesia

Animal anesthesia is classified into short-duration anesthesia and prolonged-duration anesthesia, based on its duration. Short-duration anesthesia was the intramuscular injection of 20 mg/kg Zoletil (Virbac, France), which could be maintained for approximately 10 min, and was suitable for operations such as blood collection and tissue biopsy. On the basis of short-duration anesthesia, prolonged anesthesia required intravenous injection of 3 mg/kg propofol to maintain anesthesia. The main operations performed during prolonged anesthesia were intraperitoneal drug injection, stem cell infusion, magnetic resonance imaging, and ultrasonography.

### 4.3. Determination of Toxin

In the previous study, we established the ALF monkey model with *α*-amanitin (25 *μ*g/kg bodyweight) and LPS (1 *μ*g/kg) [19]. To eliminate the impact of the LPS, in the initial pre-experiment, 20 and 40 *μ*g/kg bodyweight of *α*-amanitin without LPS were administrated in two monkeys, respectively. The monkey that received 40 *μ*g/kg *α*-amanitin died within 96 hr, while the other one survived but displayed robustly increased hepatic indices and noticeable histological changes. Thus, we decided to establish monkey models of mild and severe acute liver injury by slow infusion of 20 and 40 *μ*g/kg bodyweight of *α*-amanitin, respectively, which was modified on the basis of our previous report [10].

### 4.4. hUC-MSC Therapy

MSCs were collected from donated human umbilical cords by the Sichuan Stem Cell Bank and Sichuan New Life Stem Cell Technology, followed by culturing in serum-free medium (Stem cell 05420MesenCultTM-XF Medium, StemRD) [19]. Cultured MSCs were examined for quality control by flow cytometry checking the expression of surface biomarkers. To check the multipotent differentiation potential, osteogenic and adipogenic differentiation of hUC-MSCs were induced by culturing in a certain medium. Viral factors (hepatitis B surface antigen, hepatitis C antibody, HIV, syphilis, and cytomegalovirus), bacteria (aerobes, anaerobes, and fungi), and endotoxin were monitored. All test results were provided by the company, while hUC-MSCs with viability of over 95% at p3-5 were applied in the subsequent process.

### 4.5. Transplantation of hUC-MSCs to Rescue ALF Monkeys

Animals were randomly assigned to an intervention group (MSC+) and control group (MSC−). For MSC+ group, 1 U (1 × 10^7^) or 2 U (2 × 10^7^) of MSCs was suspended in 100 mL saline before slow peripheral infusion at 48 hr after toxin challenge. It took about 30–50 min to complete the cell transplantation with an infusion rate of 15–30 drops/min. For MSC− group, an equal volume of saline was applied at the same time point.

### 4.6. Statistical Analysis

All data were presented as mean (± SEM). Student's *t*-test was used to compare selected parameters between two groups or compare with the data at −48 hr. Survival outcomes were plotted by Kaplan–Meier curves, while the difference was determined by log-rank analysis. Data processing was performed by SPSS 24.0 and GraphPad 9.0.0 analysis software. *P* value < 0.05 was considered as statistically significant.

## 5. Conclusion

Treatment of hUC-MSCs significantly improved pathologic and survival outcomes in monkeys when the systemic inflammation was fully developed by ameliorating the activation of c-Mos, decreasing inflammatory factors, and improving liver repair. A higher dose of hUC-MSCs tended to have a better therapeutic effect. Nevertheless, we are still unable to trace the distribution, metabolism, and function of hUC-MSCs in vivo, and the underlying mechanisms need further investigation. In summary, using a nonhuman primate model, our cohort study indicates that hUC-MSCs exhibit great potential for prevention and recovery from ALF, providing a clinical solution for this lethal syndrome.

## Data Availability

The original contributions presented in the study are included in the article/supplementary material; further inquiries can be directed to the corresponding author.

## Abbreviations

ALB: Albumin
ALF: Acute liver failure
ALT: Alanine aminotransferase
APTT: Activated partial thromboplastin time
AST: Aspartate aminotransferase
BA: Blood ammonia
CCR2: C-C chemokine receptor type 2
CD: Cluster of differentiation
c-Mos: Circulating monocytes
hUC-MSCs: Human umbilical cord MSCs
IL: Interleukin
IL-1*β*: Interleukin-1 beta
IL-1RA: Interleukin-1 receptor antagonist
IL-4: Interleukin-4
IL-6: Interleukin-6
IL-10: Interleukin-10
LPS: Lipopolysaccharide
PT: Prothrombin time
TBIL: Total bilirubin
TNF-*α*: Tumor necrosis factor.

## Supplementary Materials

Supplementary Materials Supplementary material includes experimental methods for immunohistochemistry, flow cytometry, blood biochemical indexes, general information, and treatments of monkeys.

## References

[1] Stravitz R. T., Lee W. M. (2019). Acute liver failure. *The Lancet*.

[2] Wendon J., Cordoba J., Dhawan A. (2017). EASL clinical practical guidelines on the management of acute (fulminant) liver failure. *Journal of Hepatology*.

[3] Park S. J., Lim Y.-S., Hwang S. (2010). Emergency adult-to-adult living-donor liver transplantation for acute liver failure in a hepatitis B virus endemic area. *Hepatology*.

[4] Olthoff K. M., Smith A. R., Abecassis M. (2015). Defining long-term outcomes with living donor liver transplantation in North America. *Annals of Surgery*.

[5] Wennig R., Eyer F., Schaper A., Zilker T., Andresen-Streichert H. (2020). Mushroom poisoning. *Deutsches Ärzteblatt International*.

[6] Garcia J., Costa V. M., Carvalho A. (2015). *Amanita phalloides* poisoning: mechanisms of toxicity and treatment. *Food and Chemical Toxicology*.

[7] Karvellas C. J., Tillman H., Leung A. A. (2016). Acute liver injury and acute liver failure from mushroom poisoning in North America. *Liver International*.

[8] Zhou P., Xia J., Guo G. (2012). A *Macaca mulatta* model of fulminant hepatic failure. *World Journal of Gastroenterology*.

[9] Jedicke N., Struever N., Aggrawal N. (2014). *α*-1-antitrypsin inhibits acute liver failure in mice. *Hepatology*.

[10] Guo G., Zhu Y., Wu Z. (2018). Circulating monocytes accelerate acute liver failure by IL-6 secretion in monkey. *Journal of Cellular and Molecular Medicine*.

[11] Rolando N., Wade J., Davalos M., Wendon J., Philpott-Howard J., Williams R. (2000). The systemic inflammatory response syndrome in acute liver failure. *Hepatology*.

[12] Watanabe Y., Tsuchiya A., Seino S. (2019). Mesenchymal stem cells and induced bone marrow-derived macrophages synergistically improve liver fibrosis in mice. *Stem Cells Translational Medicine*.

[13] Elmahallawy E. K., Mohamed Y., Abdo W., Yanai T. (2020). Melatonin and mesenchymal stem cells as a key for functional integrity for liver cancer treatment. *International Journal of Molecular Sciences*.

[14] Cao H., Yang J., Yu J. (2012). Therapeutic potential of transplanted placental mesenchymal stem cells in treating Chinese miniature pigs with acute liver failure. *BMC Medicine*.

[15] He Y., Guo X., Lan T. (2021). Human umbilical cord-derived mesenchymal stem cells improve the function of liver in rats with acute-on-chronic liver failure via downregulating Notch and Stat1/Stat3 signaling. *Stem Cell Research and Therapy*.

[16] Xie Q., Liu R., Jiang J. (2020). What is the impact of human umbilical cord mesenchymal stem cell transplantation on clinical treatment?. *Stem Cell Research and Therapy*.

[17] Yin F., Wang W.-Y., Jiang W.-H. (2019). Human umbilical cord mesenchymal stem cells ameliorate liver fibrosis in vitro and in vivo: from biological characteristics to therapeutic mechanisms. *World Journal of Stem Cells*.

[18] Shao M., Xu Q., Wu Z. (2020). Exosomes derived from human umbilical cord mesenchymal stem cells ameliorate IL-6-induced acute liver injury through miR-455-3p. *Stem Cell Research and Therapy*.

[19] Guo G., Zhuang X., Xu Q. (2019). Peripheral infusion of human umbilical cord mesenchymal stem cells rescues acute liver failure lethality in monkeys. *Stem Cell Research and Therapy*.

[20] Goren A., Dahan N., Goren E., Baruch L., Machluf M. (2009). Encapsulated human mesenchymal stem cells: a unique hypoimmunogenic platform for long-term cellular therapy. *The FASEB Journal*.

[21] Fan C.-G., Zhang Q.-J., Zhou J.-R. (2011). Therapeutic potentials of mesenchymal stem cells derived from human umbilical cord. *Stem Cell Reviews and Reports*.

[22] Li Y., Wu Q., Wang Y. (2018). Novel spheroid reservoir bioartificial liver improves survival of nonhuman primates in a toxin-induced model of acute liver failure. *Theranostics*.

[23] Kayagaki N., Stowe I. B., Lee B. L. (2015). Caspase-11 cleaves gasdermin D for non-canonical inflammasome signalling. *Nature*.

[24] Antoniades C. G., Quaglia A., Taams L. S. (2012). Source and characterization of hepatic macrophages in acetaminophen-induced acute liver failure in humans. *Hepatology*.

[25] Possamai L. A., Thursz M. R., Wendon J. A., Antoniades C. G. (2014). Modulation of monocyte/macrophage function: a therapeutic strategy in the treatment of acute liver failure. *Journal of Hepatology*.

[26] Cardoso F. S., Gottfried M., Tujios S., Olson J. C., Karvellas C. J., for the US Acute Liver Failure Study Group (2018). Continuous renal replacement therapy is associated with reduced serum ammonia levels and mortality in acute liver failure. *Hepatology*.

[27] Kim J.-H., Jo C. H., Kim H.-R., Hwang Y.-I. (2018). Comparison of immunological characteristics of mesenchymal stem cells from the periodontal ligament, umbilical cord, and adipose tissue. *Stem Cells International*.

[28] Hu C., Wu Z., Li L. (2020). Mesenchymal stromal cells promote liver regeneration through regulation of immune cells. *International Journal of Biological Sciences*.

[29] Ju C., Tacke F. (2016). Hepatic macrophages in homeostasis and liver diseases: from pathogenesis to novel therapeutic strategies. *Cellular and Molecular Immunology*.

[30] Melgar-Lesmes P., Edelman E. R. (2015). Monocyte-endothelial cell interactions in the regulation of vascular sprouting and liver regeneration in mouse. *Journal of Hepatology*.

[31] Wang J., Kubes P. (2016). A reservoir of mature cavity macrophages that can rapidly invade visceral organs to affect tissue repair. *Cell*.

[32] Yun J.-W., Ahn J. H., Kwon E. (2016). Human umbilical cord-derived mesenchymal stem cells in acute liver injury: hepatoprotective efficacy, subchronic toxicity, tumorigenicity, and biodistribution. *Regulatory Toxicology and Pharmacology*.

[33] Wang L., Huang S., Li S. (2019). Efficacy and safety of umbilical cord mesenchymal stem cell therapy for rheumatoid arthritis patients: a prospective phase I/II study. *Drug Design, Development and Therapy*.

[34] Deng W.-S., Ma K., Liang B. (2020). Collagen scaffold combined with human umbilical cord-mesenchymal stem cells transplantation for acute complete spinal cord injury. *Neural Regeneration Research*.

[35] Shu L., Niu C., Li R. (2020). Treatment of severe COVID-19 with human umbilical cord mesenchymal stem cells. *Stem Cell Research and Therapy*.

[36] Li J., Xu S. Q., Zhao Y. M., Yu S., Ge L. H., Xu B. H. (2018). Comparison of the biological characteristics of human mesenchymal stem cells derived from exfoliated deciduous teeth, bone marrow, gingival tissue, and umbilical cord. *Molecular Medicine Reports*.

[37] Wang D., Zhang H., Liang J. (2018). A long-term follow-up study of allogeneic mesenchymal stem/stromal cell transplantation in patients with drug-resistant systemic lupus erythematosus. *Stem Cell Reports*.

[38] Fang X., Liu L., Dong J. (2018). A study about immunomodulatory effect and efficacy and prognosis of human umbilical cord mesenchymal stem cells in patients with chronic hepatitis B-induced decompensated liver cirrhosis. *Journal of Gastroenterology and Hepatology*.

