# Supplementary material for: Peripheral Injection of hUC-MSCs in the Treatment of Acute Liver Failure: A Pre-Clinical Cohort Study in Rhesus Monkeys
Source: Stem Cells Int. 2024 Jul 16;2024:4654912. doi: 10.1155/2024/4654912 (PMC11265939; doi:10.1155/2024/4654912)
Supplement: Supplementary Materials — Supplementary material includes experimental methods for immunohistochemistry, flow cytometry, blood biochemical indexes, general information, and treatments of monkeys. [file 4654912.f1.docx]

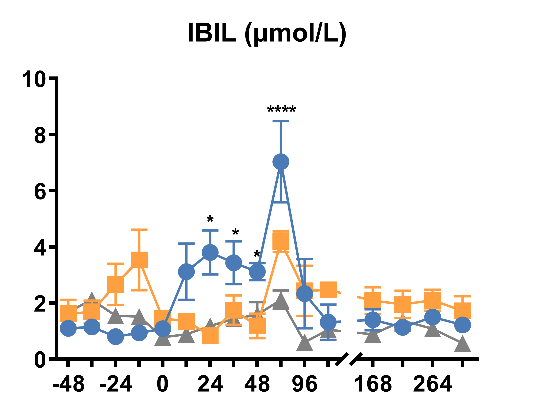

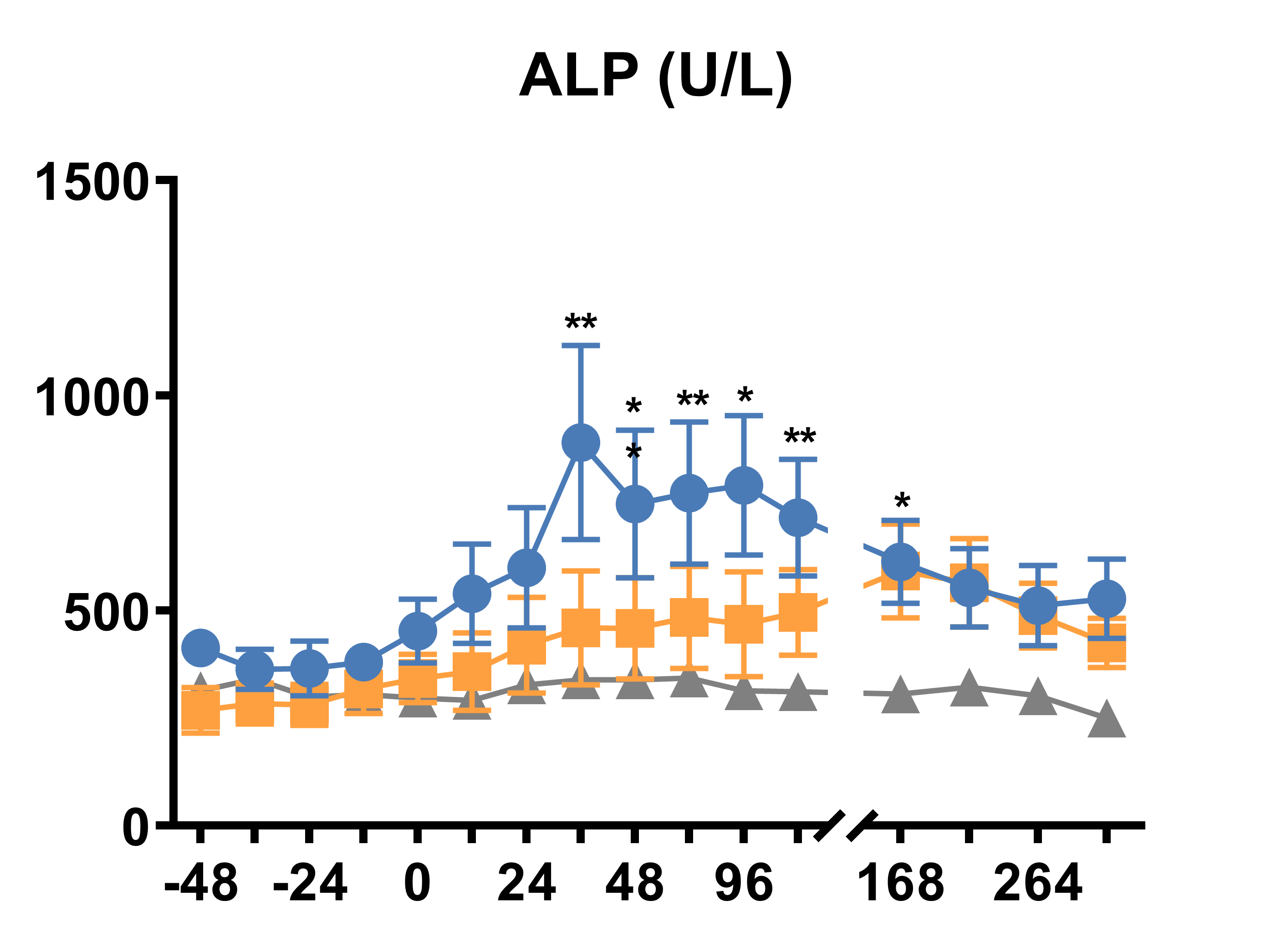

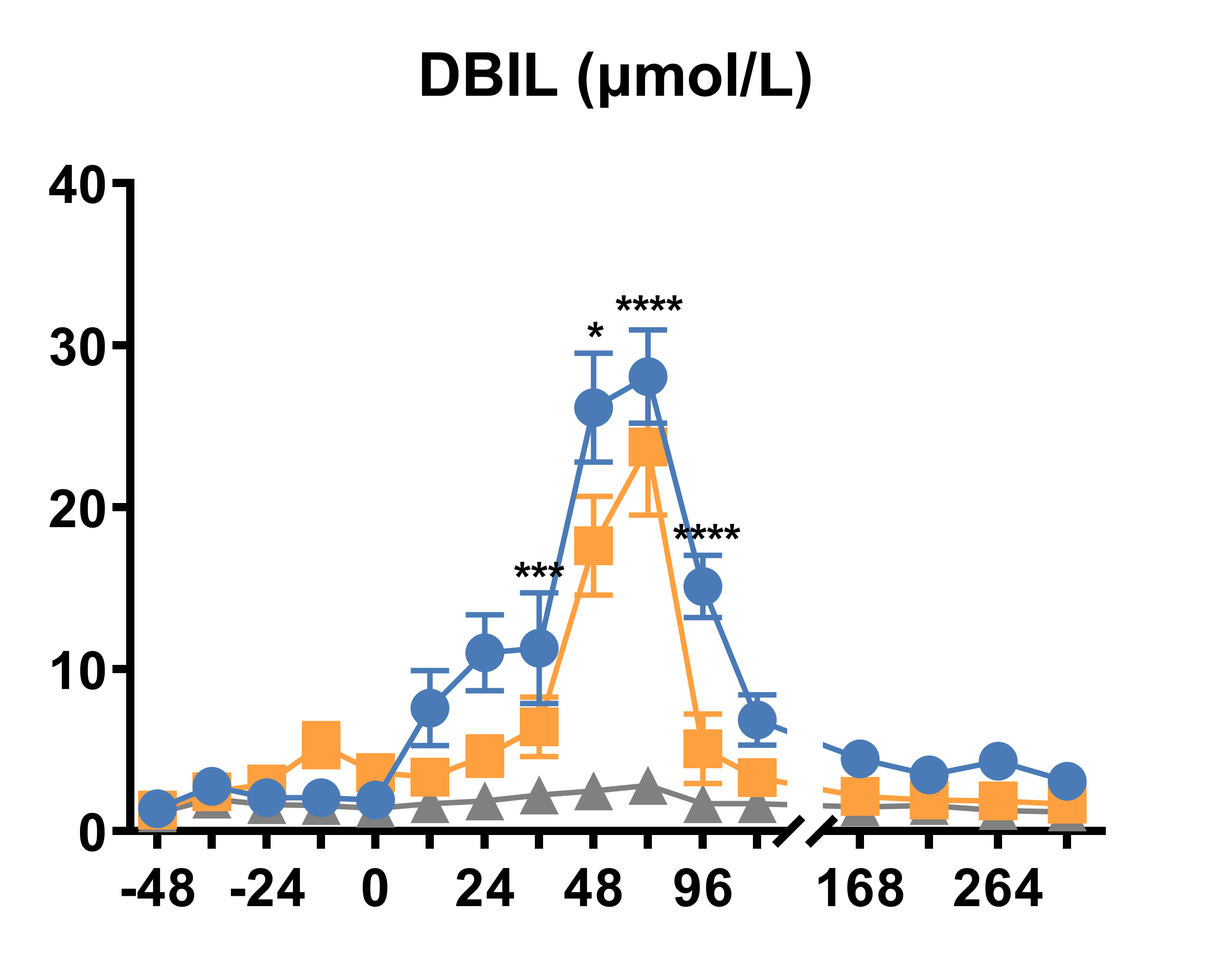

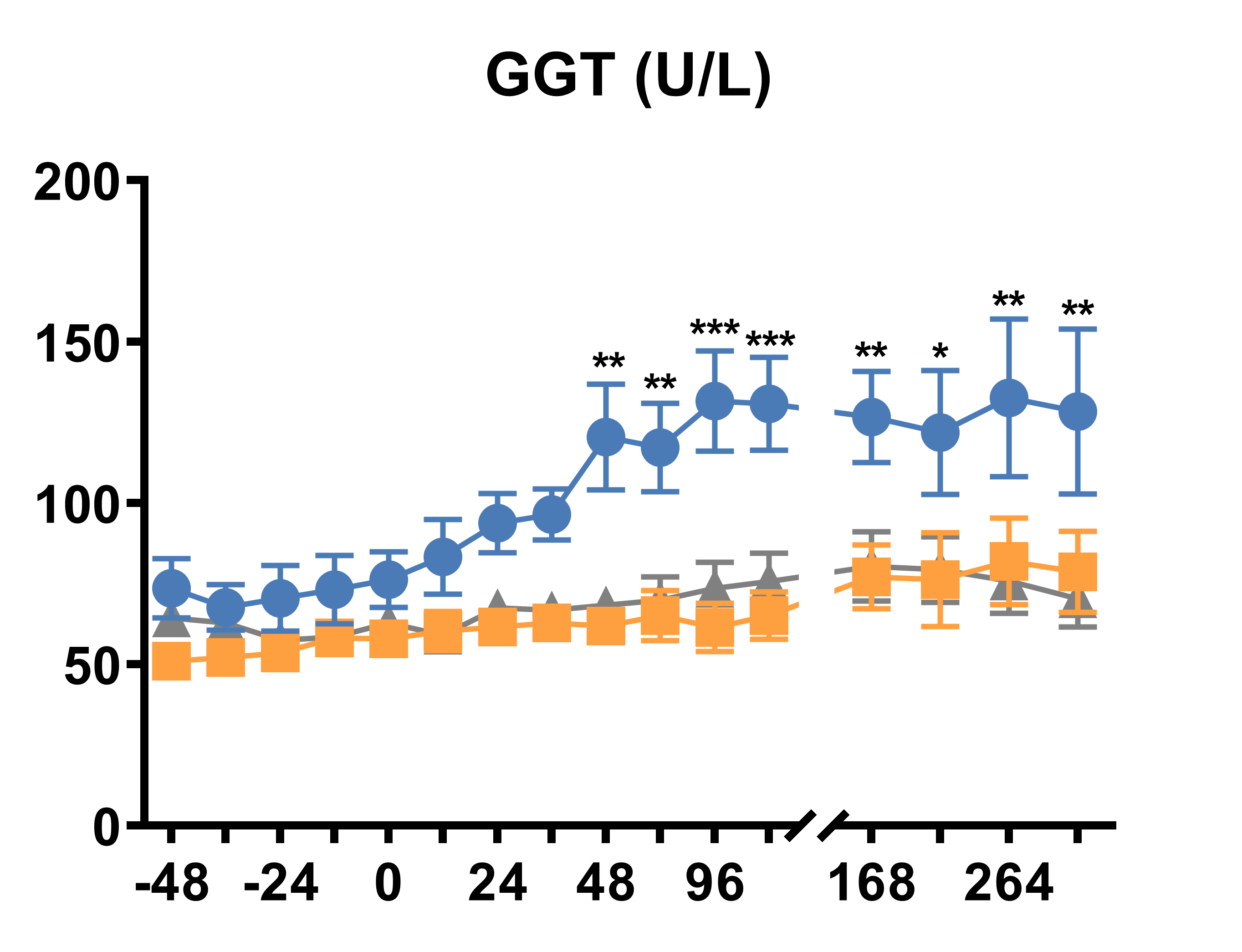

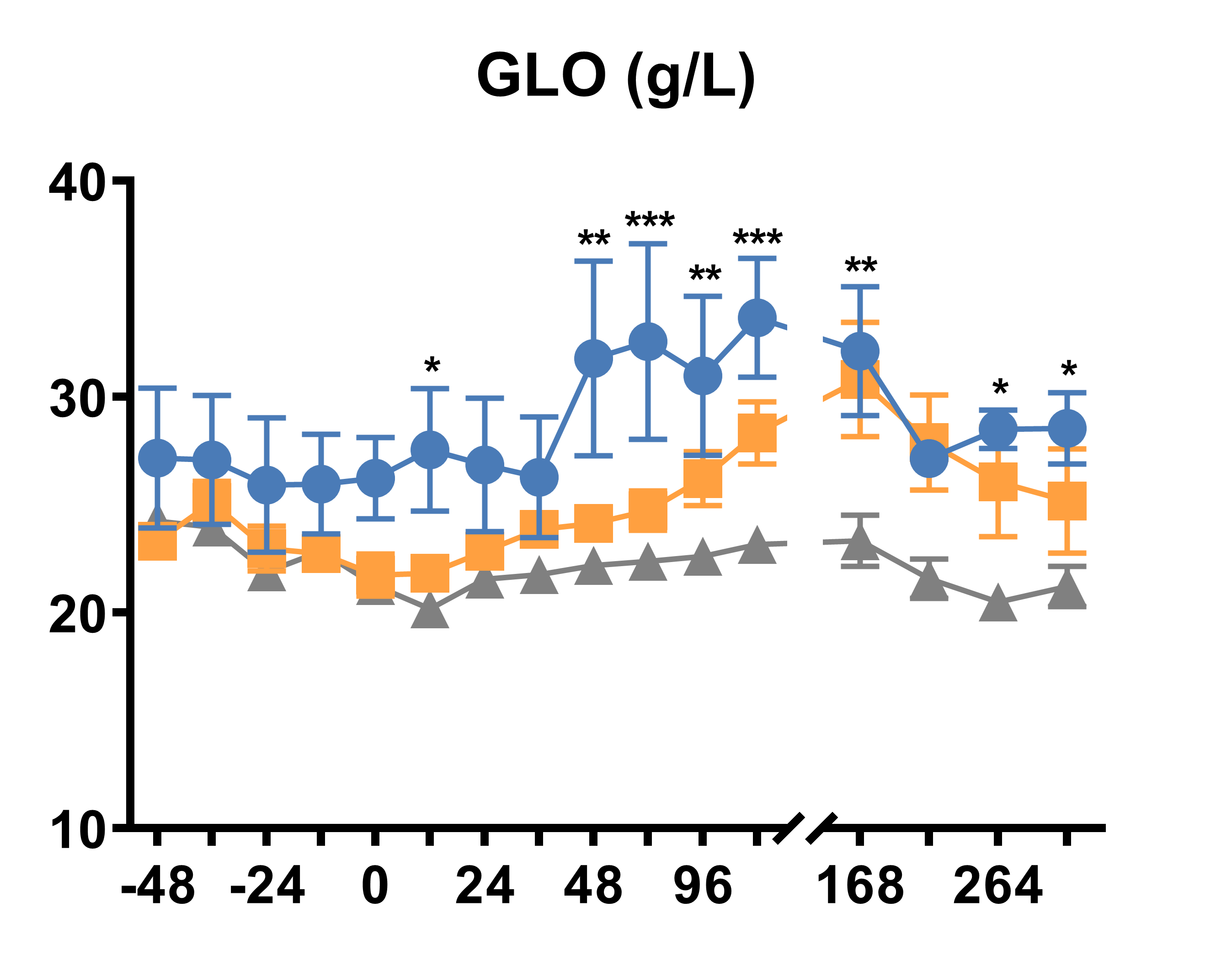

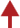

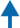

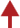

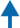

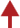

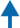

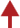

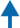

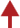

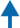

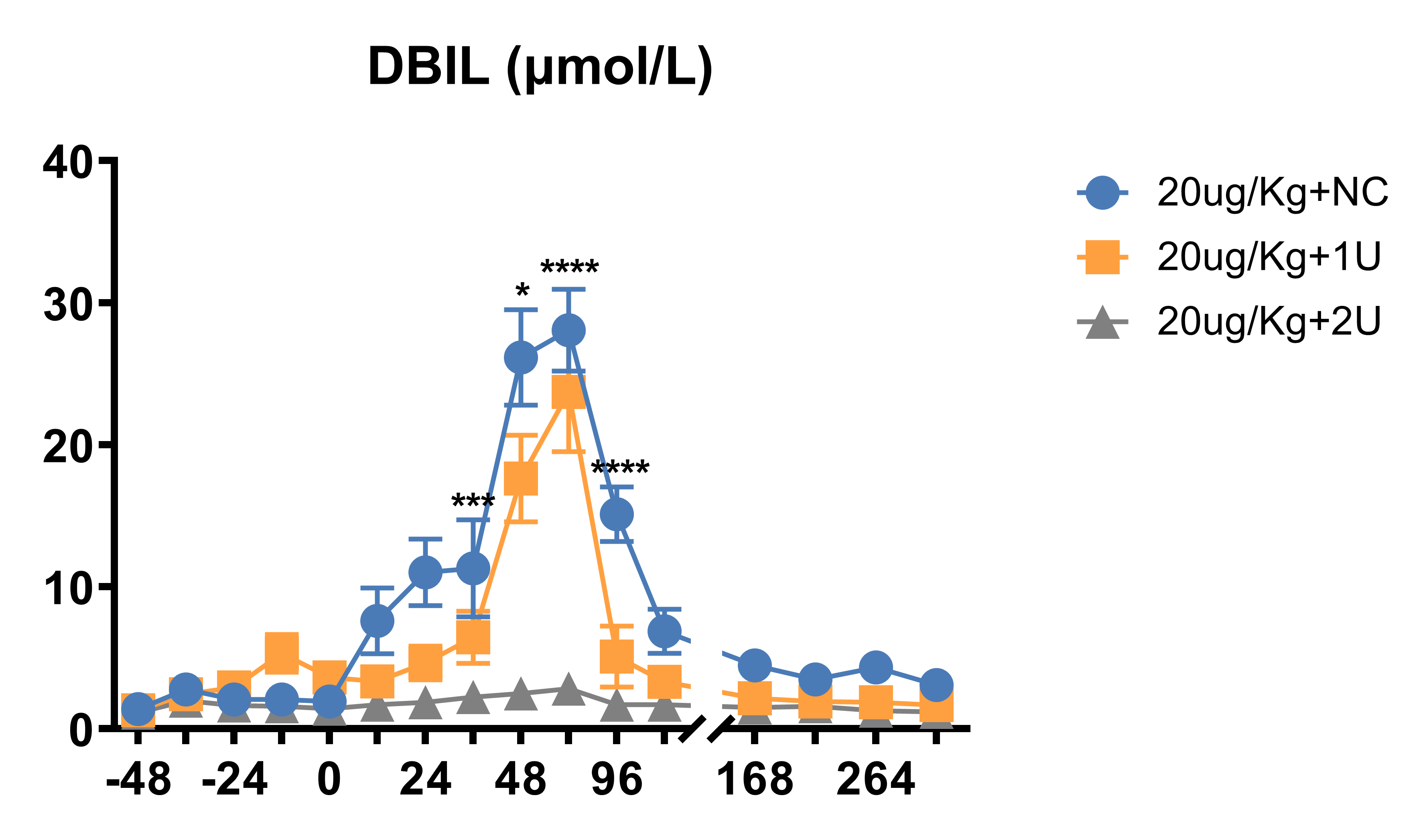

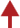

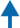


**Saline**

**1-U MSC**

**2-U MSC**

**Toxin injection**

**hUC-MSC infusion**

**Figure S1. Peripheral delivery of hUC-MSCs ameliorates liver histology and hepatic indices in 20 μg/kg of α-amanitin group.** Biochemical assay of hepatic indices: direct bilirubin (DBIL), indirect bilirubin (IBIL), alkaline phosphatase (ALP), glutamyl transpeptidase (GGT), globulin (GLO). Error bars, SEM. *P < 0.05, **P < 0.01, and ***P < 0.001, compared with the data at 48 h.

**Figure S2. Peripheral delivery of hUC-MSCs ameliorates liver histology and hepatic indices in 40 μg/kg of α-amanitin group.** Biochemical assay of hepatic indices: direct bilirubin (DBIL), indirect bilirubin (IBIL), alkaline phosphatase (ALP), glutamyl transpeptidase (GGT), total bile acid (TBA). Error bars, SEM. *P < 0.05, **P < 0.01, and ***P < 0.001, compared with the data at 48 h.


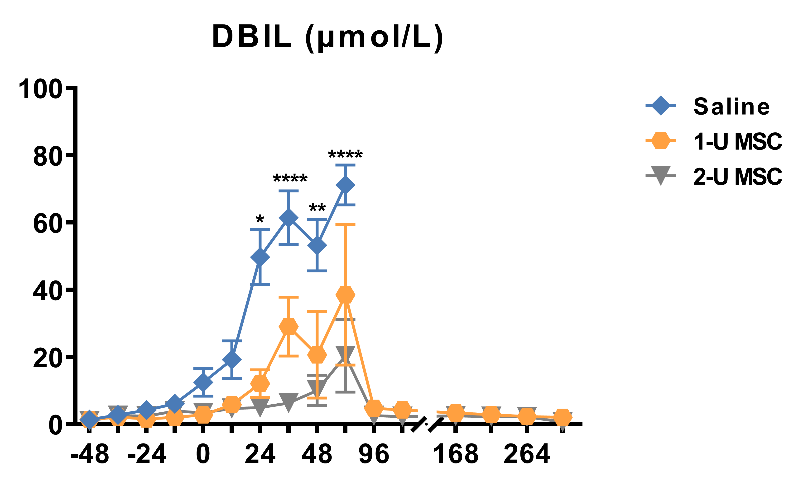

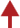

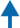

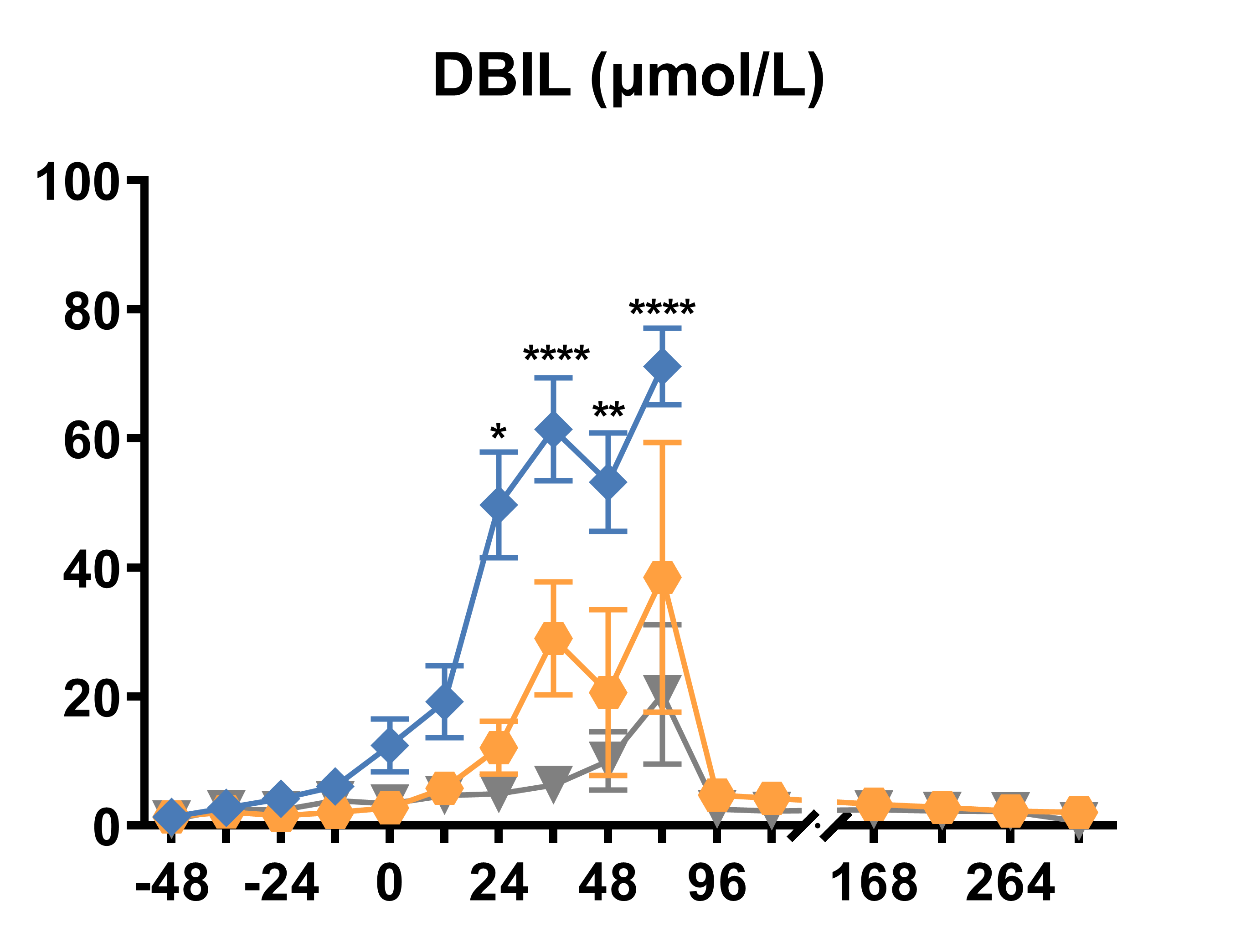

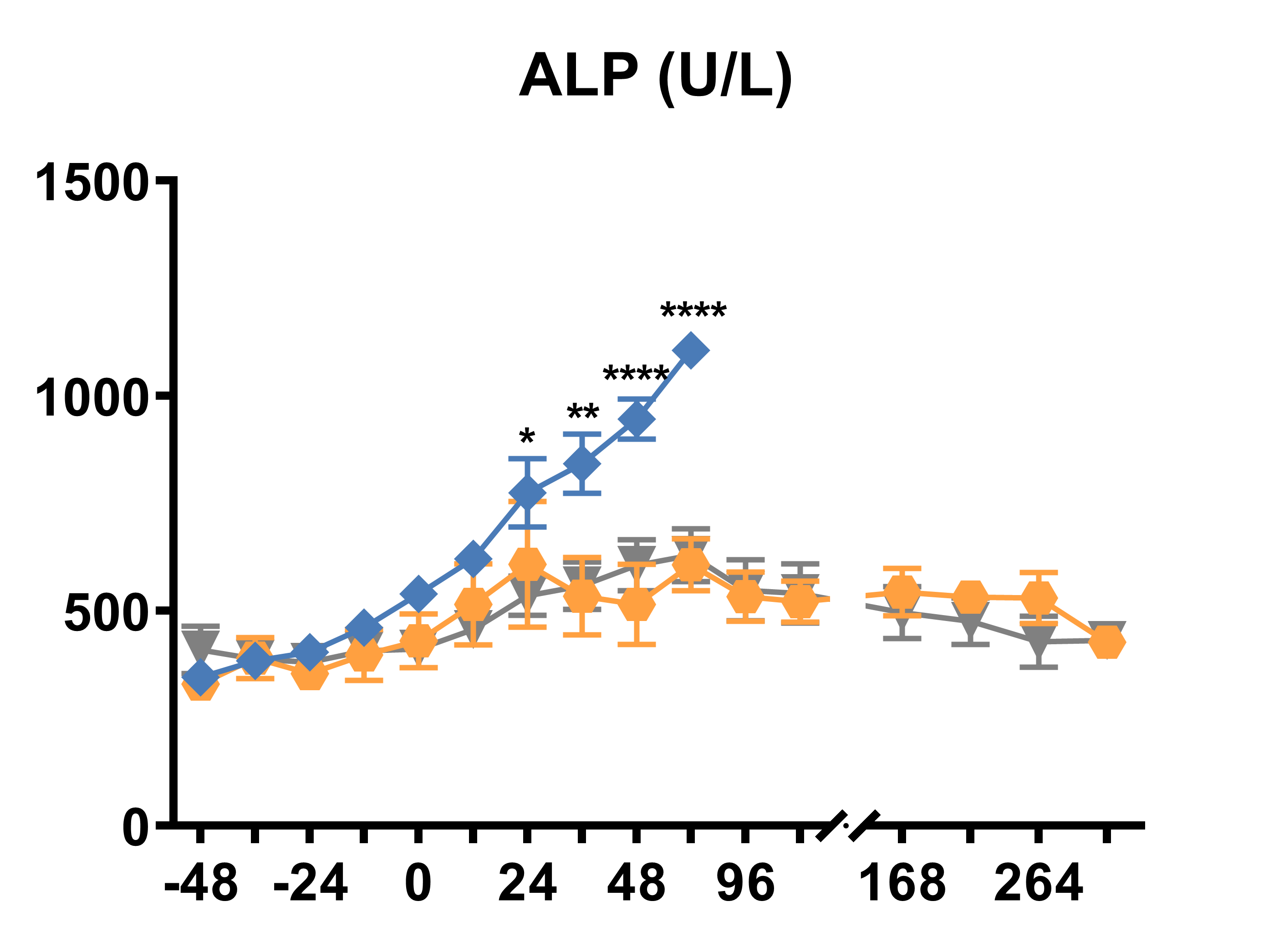

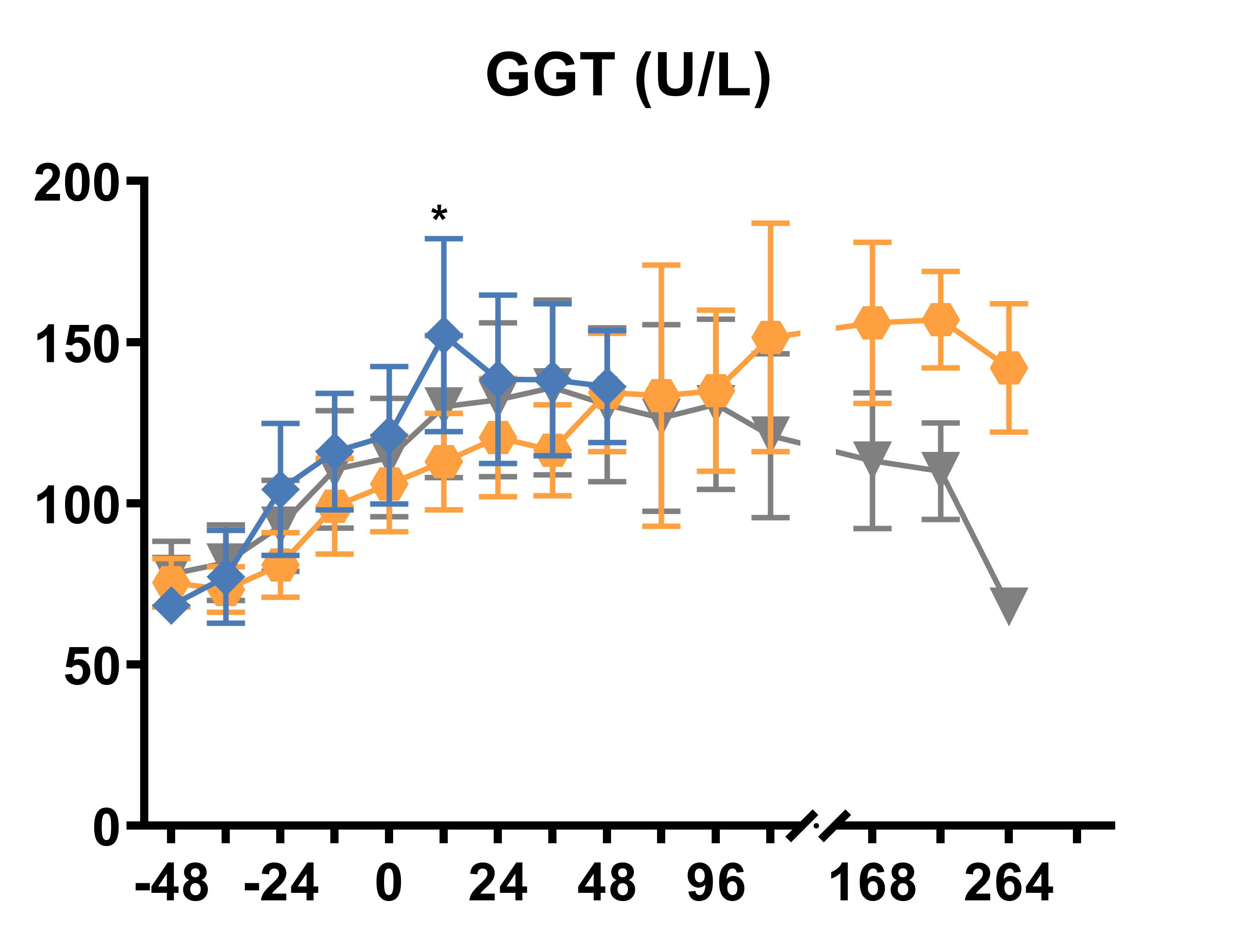

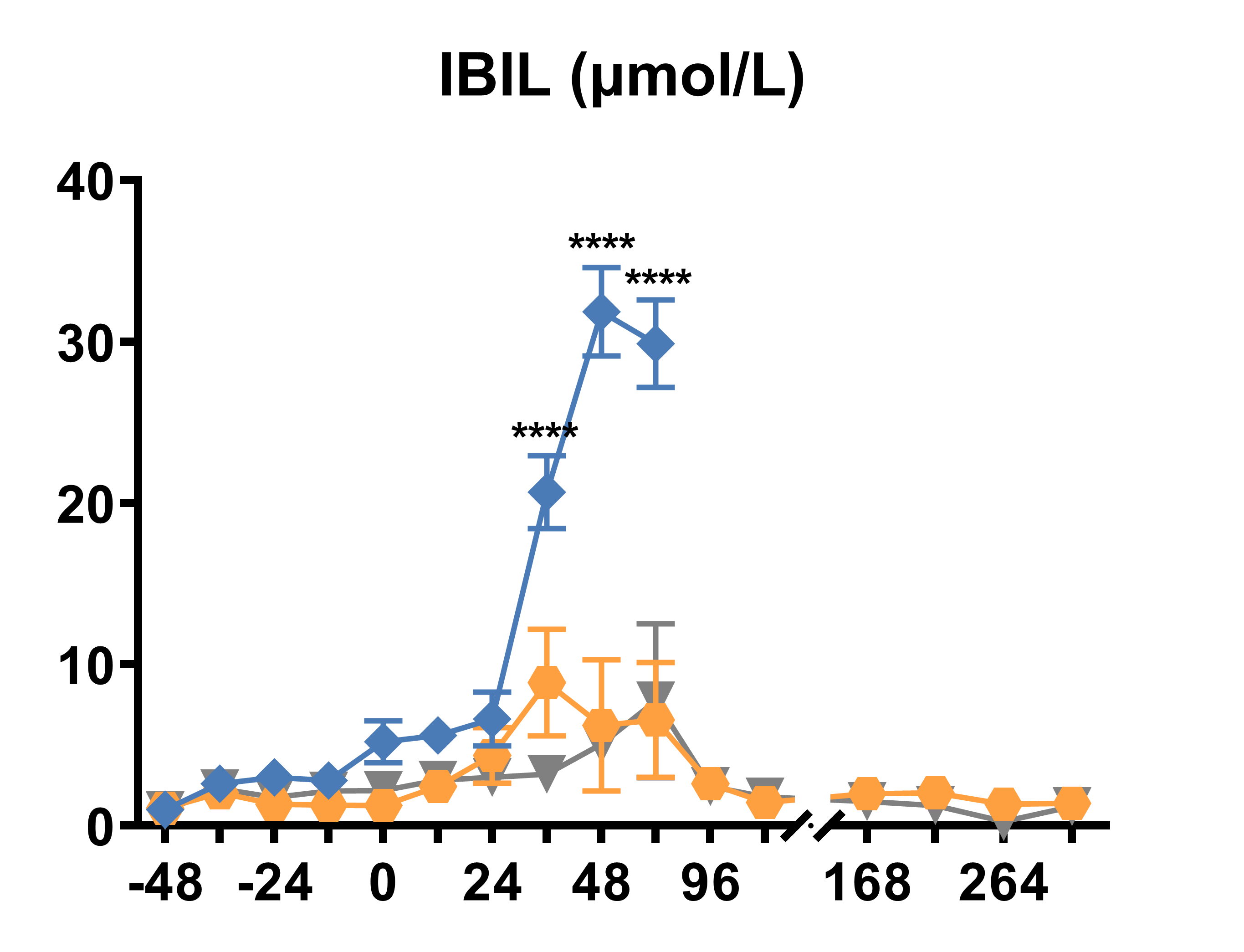

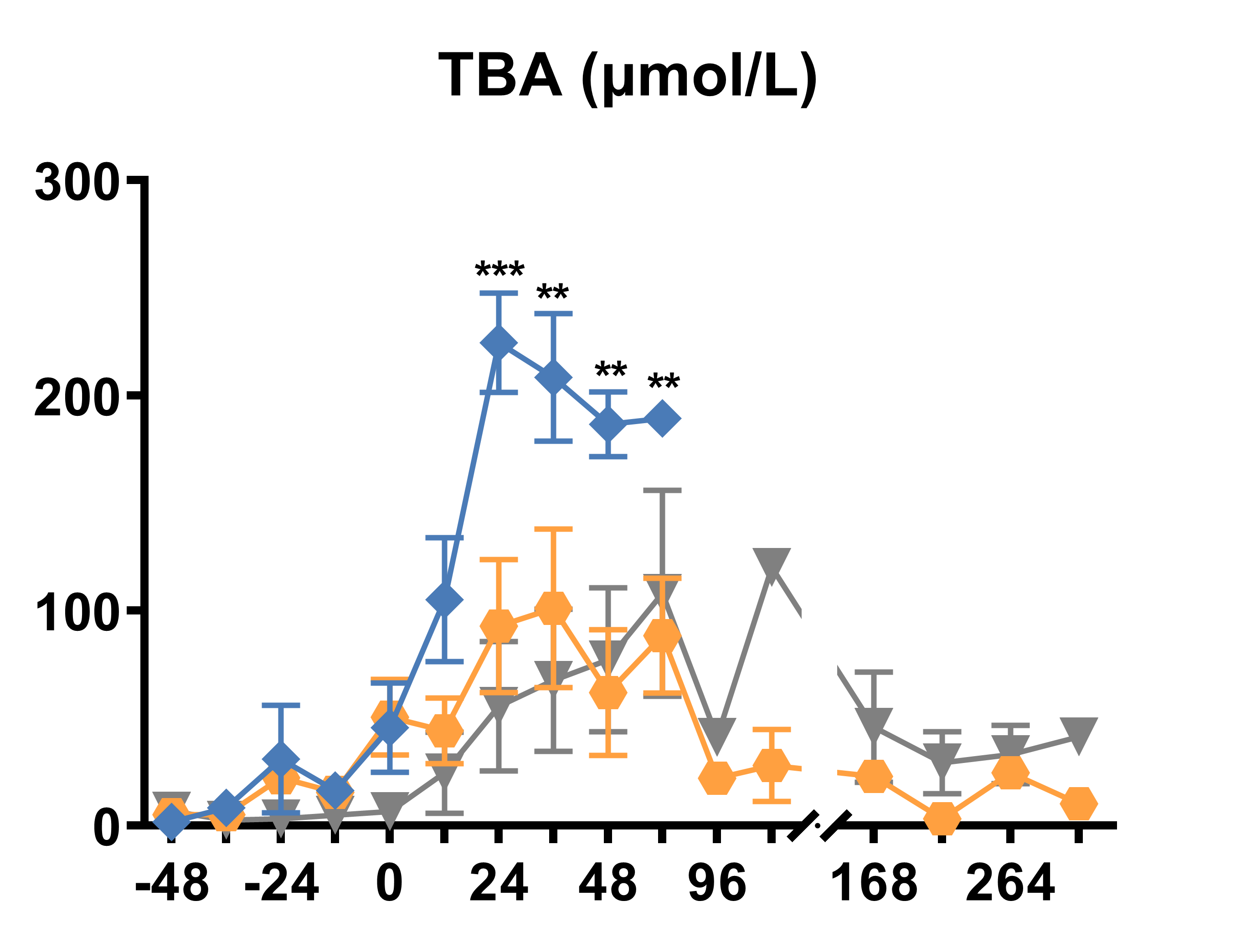

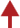

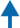

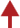

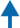

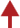

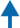

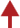

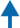

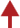

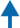


**Saline**

**1-U MSC**

**2-U MSC**

**Toxin injection**

**hUC-MSC infusion**

Supplement methods

Immunohistochemistry: The monkey liver specimens were made into 4μm frozen section for oil red O staining and fluorescence immunohistochemistry. For H&E staining, tissues were fixed in 4% neutral buffered formalin for 48 hours and embedded in paraffin, sectioned at 4 μm and staining with hematoxylin and eosin. Immunohistochemistry and immunofluo- rescence of Ki67 antibody (1:100, Thermo Fisher, Grand Island, NY) were used to measure cell proliferation.

Flow cytometry: Monoclonal antibodies against CD14, CD16 and CCR2 (BD Biosciences) were used to determine monocyte subsets and CCR2 expression. Flow cytometry was performed on a Beckman Coulter FC500 and analysed using a Kaluza v1.20 software (Beckman Coulter, Fullerton, CA) or CXP analysis software.

Blood biochemical indexes: Blood serum and plasma was isolated by centrifugation at 1500 g for 10 minutes at 4°C for biochemical evaluation and cytokine quantification. All the hepatic parameters were analysed in a standard clinical laboratory in West China Hospital, Sichuan University.

Cytokines analysis: A MILLIPLEX MAP Human Cytokine/Chemokine kit (Millipore, Billerica, MA, USA; cat # HCYTOMAG-60 K) was used to quantify the levels of cytokines, chemokines, and growth factors in human cell supernatants on a Luminex 200 System (Millipore) according to the manufacturer’s instructions.

Supplement Table 1

General information and treatments of Monkeys

| Monkey ID | Sex（F/M） | Age (y) | | Weight (kg) | | Toxin (μg/kg) | | hUC-MSC (U) | | Death Time | |
| --- | --- | --- | --- | --- | --- | --- | --- | --- | --- | --- | --- |
| 13537 | M | 5 | 6.4 | | 40 | | N | | 72h | |  |
| 13641 | M | 5 | 6.1 | | 40 | | N | | 84h | |  |
| 14005 | M | 4.5 | 5.86 | | 40 | | N | | 84h | |  |
| 13451 | M | 5 | 6.8 | | 40 | | N | | 24h | |  |
| 13671 | M | 5 | 6.1 | | 40 | | N | | N | |  |
| 14651 | M | 4 | 5.86 | | 40 | | N | | 84h | |  |
| 13629 | M | 5 | 8.3 | | 40 | | N | | 12h | |  |
| 13329 | M | 5 | 7.14 | | 40 | | 1 | | 86h | |  |
| 13713 | M | 5 | 6.64 | | 40 | | 1 | | N | |  |
| 13521 | M | 5 | 6.6 | | 40 | | 1 | | 60h | |  |
| 13847 | M | 5 | 7 | | 40 | | 1 | | 84h | |  |
| 14077 | M | 4 | 5.8 | | 40 | | 1 | | N | |  |
| 14639 | M | 4 | 5.5 | | 40 | | 1 | | 60h | |  |
| 13579 | M | 5 | 6.3 | | 40 | | 2 | | 84h | |  |
| 13783 | M | 5 | 6.6 | | 40 | | 2 | | 84h | |  |
| 14257 | M | 4 | 6.5 | | 40 | | 2 | | N | |  |
| 14263 | M | 4 | 5.8 | | 40 | | 2 | | 48h | |  |
| 13101 | M | 5 | 7.8 | | 40 | | 2 | | N | |  |
| 13755 | M | 5 | 7.8 | | 40 | | 2 | | N | |  |
| 14177 | M | 4 | 4.6 | | 20 | | N | | N | |  |
| 14065 | M | 4 | 4.8 | | 20 | | N | | N | |  |
| 14509 | M | 4 | 5.8 | | 20 | | N | | N | |  |
| 13813 | M | 5 | 8.3 | | 20 | | N | | N | |  |
| 14277 | M | 4 | 6 | | 20 | | N | | N | |  |
| 14613 | M | 4 | 6.2 | | 20 | | N | | N | |  |
| 14043 | M | 4 | 4.9 | | 20 | | 2 | | N | |  |
| 14201 | M | 4 | 4.8 | | 20 | | 2 | | N | |  |
| 14223 | M | 4 | 5 | | 20 | | 2 | | N | |  |
| 14355 | M | 4 | 5 | | 20 | | 2 | | N | |  |
| 14385 | M | 4 | 5 | | 20 | | 2 | | N | |  |
| 14571 | M | 4 | 4.6 | | 20 | | 2 | | N | |  |
| 15115 | M | 3 | 4.9 | | 20 | | 1 | | N | |  |
| 15169 | M | 3 | 4.6 | | 20 | | 1 | | N | |  |
| 15317 | M | 3 | 4.7 | | 20 | | 1 | | N | |  |
| 13331 | M | 5 | 7.2 | | 20 | | 1 | | N | |  |
| 13785 | M | 5 | 4.8 | | 20 | | 1 | | N | |  |
| 14085 | M | 4 | 4.8 | | 20 | | 1 | | N | |  |
